# Characterization of the complete chloroplast genome of Korean endemic, *Habenaria cruciformis* (Orchidaceae)

**DOI:** 10.1080/23802359.2020.1812448

**Published:** 2020-08-31

**Authors:** Changkyun Kim, Hoang Dang Khoa Do, Joonhyung Jung, Dong-Kap Kim, Joo-Hwan Kim

**Affiliations:** a Department of Life Science, Gachon University, Seongnam, Republic of Korea; b Nguyen Tat Thanh Hi-Tech Institute, Nguyen Tat Thanh University, Ho Chi Minh City, Viet Nam; c Forest Biodiversity Division, Korea National Arboretum, Pocheon, Republic of Korea

**Keywords:** Chloroplast genome, endemic, *Habaneria cruciformis*, Orchidaceae, phylogenomic analyses

## Abstract

*Habenaria cruciformis* (Orchidaceae), endemic to South Korea, is a perennial herb and its local population sizes are declined because of the destruction caused by human activity and the invasion of exotic species in their habitats. Here, we report the complete chloroplast (cp) genome sequence of *H. cruciformis*, which will provide valuable information for its biological conservation and future studies for the cp genome evolution of endemic plants on the Korean Peninsula. The cp genome of *H. cruciformis* is 155,708 bp in length, containing a large single-copy region of 85,131 bp and a small single-copy region of 17,659 bp which are separated by a pair of inverted repeats of 26,459 bp. The *H. cruciformis* cp genome encodes 131 genes, of which 113 are unique, including 79 protein-coding genes, 30 tRNA genes, and 4 rRNA genes. The overall GC content is 36.6%, which is consistent with the *Habenaria* species previously reported. Our phylogenomic analyses identified the sister relationship between *H. cruciformis* and *H. linearfolia* in the genus.

Endemic species are important for understanding speciation mechanisms and biogeographic history in a certain geographical region because the distribution of species is closely related to their evolutionary history (Cox and Moore 2010). Many studies have been recently conducted to identify the characteristics of chloroplast (cp) genome and develop useful molecular markers in order to determine speciation patterns and genetic diversity for endemic species (Cheon et al. 2017; Kim et al. 2019).

*Habenaria cruciformis* (Orchidaceae) is an endemic in South Korea and found in wetland under mountains or in grasslands (Lee and Choi 2006). This species is morphologically similar to *H. linearfolia* but differ in spur length (13–16 mm vs. 23–63 mm) and color and shape of lateral lobes (green and recurved vs. white and spreading) (Lee 2007). Despite the morphological distinctiveness of *H. cruciformis*, its genetic identity and sister group within the genus have not been addressed. The local population sizes of *H. cruciformis* are declined because of destruction either directly or indirectly caused by human activity and the invasion of exotic species in their habitats (National Institute of Biological Resources 2012). Here, we determined the cp genome sequences of *H. cruciformis* to provide useful genetic information in phylogenetic relationship, phylogeographic history, and conservation of the Korean endemic plants.

An individual of *H. cruciformis* was collected from Jangneung wetland, Yeongwol-gun, Gangwon-do, South Korea. The voucher specimen was deposited at the herbarium of Gachon University (accession number: GCU190036385). Total genomic DNA was extracted from silica gel-dried leaf tissues using a DNeasy Plant Mini Kit (Qiagen, Valencia, California, USA). Genomic DNA was used for sequencing using an Illumina MiSeq Sequencer (Illumina, San Diego, CA, USA). After trim of low-quality reads and adapters, the raw reads (10,024,910) were aligned to the reference cp genomes of *H. radiata* (GenBank no. NC035834) and *H. pantlingiana* (NC026775). Reads were then reassembled *de novo* with no mismatches and gaps to generate contigs. The raw reads were realigned to these contigs with no mismatches and gaps and with 100 iterations. Finally, all contigs were concatenated into a circular map and a few regions with low-depth coverage were confirmed using PCR-based Sanger sequencing. The annotation of cp genome was performed using DOGMA (https://dogma.ccbb.utexas.edu; Wyman et al. 2004) and tRNAscan-SE v.2.0 (Lowe and Chan 2016). A circular cp genome map was drawn using OGDRAW v.1.2 (http://ogdraw.mpimp-golm.mpg.de; Lohse et al. 2013). The complete cp genome sequence of *H. cruciformis* was submitted to GenBank under accession number MT863537.

The complete cp genome sequence of *H. cruciformis* was 155,708 bp in length and showed a typical quadripartite structure, consisting of the large single-copy (85,131 bp) and small single-copy (17,659 bp) separated by a pair of inverted repeat (26,459 bp). The *H. cruciformis* cp genome encoded 131 predicted functional genes, of which 113 were unique and 18 duplicated in the IR regions. The unique genes comprised 79 protein-coding genes, 30 tRNA genes, and 4 rRNA genes. Duplicated genes included six protein-coding genes, eight tRNA genes, and four rRNA genes. Twelve protein-coding genes and six tRNA genes contained one or two introns. The overall GC content of *H. cruciformis* cp genome was 36.6%, and in the LSC, SSC, and IR regions were 34.2, 29.0, and 43.0%, respectively. Compared with the previously reported cp genomes of *Habenaria* species, *H. cruciformis* was quite similar in terms of gene content, gene order, and GC content.

To determine phylogenetic relationships, in addition to *H. cruciformis*, we included three *Habenaria* species (*H. linearfolia*, *H. pantilingiana*, and *H. radiata*). We also included three species (*Platanthera chlorantha*, *P. japonica*, *Dactylorhiza majalis*) from Orchideae and three species (*Anoectochilus emeiensis*, *Ludisia discolor*, *Goodyera fumata*) from Cranichideae of Orchidoideae as outgroups. Sequence alignment of the cpDNA coding regions was performed in MAFFT v.7 (Katoh et al. 2019) followed by manual adjustment. The phylogenetic reconstruction was performed with maximum parsimony (MP) and Bayesian inference (BI) methods. Most parsimonious trees were searched with a heuristic algorithm in PAUP v.4.0b10 (Swofford 2002). A BI phylogram was reconstructed using MrBayes v.3.2 (Ronquist et al. 2012) with the following parameters: nst = 6, rates = invgamma, ngen = 1,000,000, samplefreq = 1000, burn-in = 25%. Bootstrap support (BS; 10,000 replicates) and posterior probability (PP) were calculated to estimate robustness for each clade. Among the 59,841 characters of the combined cpDNA dataset, 3,853 (6.4%) were variable and 2,393 (4.0%) were parsimony-informative. Both MP and BI trees were identical in topology (Figure 1). The monophyly of *Habenaria* was strongly supported (BS = 100, PP = 1.00). Within the genus, the sister of *H. cruciformis* was *H. linearfolia* with strong support (BS = 100, PP = 1.00).

**Figure 1.**
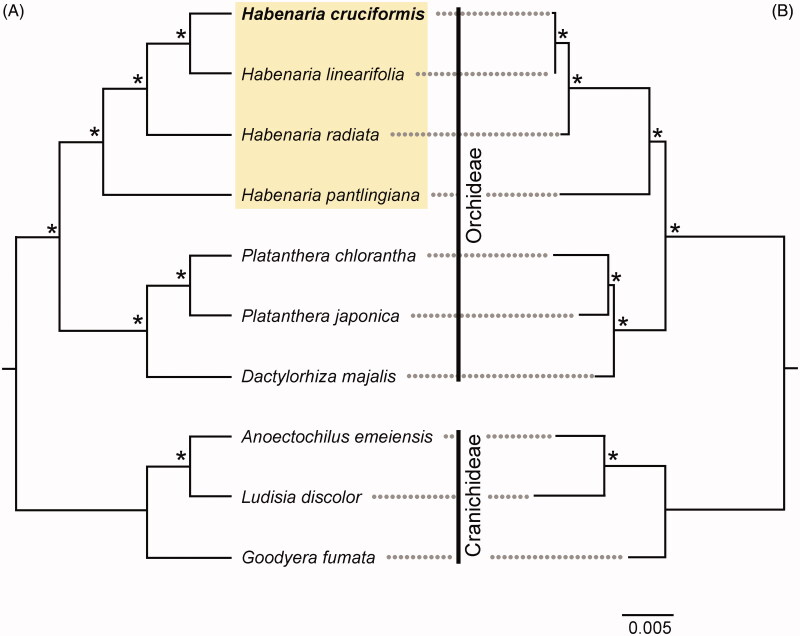
Phylogenetic trees resulting from (A) maximum parsimony (MP) and (B) Bayesian inference (BI) analyses of chloroplast 76 protein coding genes. Four *Habenaria* species are highlighted in yellow. Asterisks near nodes indicate bootstrap support = 100% for MP analysis and posterior probability = 1.00 for BI analysis. The bar represents 0.005 nucleotide substitution per site. GenBank accession numbers: *Anoectochilus emeiensis*: NC033895; *Dactylorhiza majalis*: NC044644; *Goodyera fumata*: NC026773; *Habenaria cruciformis*: MT863537; *Habenaria linearfolia*: MT863538; *Habenaria pantilingiana*: NC026775; *Habenaria radiata*: NC035834; *Platanthera chlorantha*: NC044626; *Platanthera japonica*: NC037440; *Ludisia discolor*: NC030540.

Our complete cp genome data of *H. cruciformis* may be useful in assessing the genetic diversity, genetic differentiation, and phylogeographic history of *H. cruciformis*, thereby providing a guideline for conservation. Also, it may contribute to a better understanding of the evolution of Orchidaceae.

## Data Availability

The data that supported the findings of the study are openly available in GenBank of NCBI at https://www.ncbi.nlm.nih.gov, reference number MT863537.
